# Editorial: The dynamics of environmental stresses and seed physiology: a complex interaction in plant systems

**DOI:** 10.3389/fpls.2026.1881003

**Published:** 2026-05-26

**Authors:** Vishal Varshney, Rama Shankar, Prafull Salvi

**Affiliations:** 1 Institut de Biologie Moléculaire des Plantes du CNRS (IBMP-CNRS), Strassbourg, France; 2 Department of Pediatrics and Human Development, College of Human Medicine, Michigan State University, Grand Rapids, MI, United States; 3 Agricuture Biotechnology Department, Biotechnology Research and Innovation Council (BRIC)-National Agri-Food and Biomanufacturing Institute, Punjab, India

**Keywords:** agriculture, environmental stresses, germination, global warming, multi-omics, seed longevity and viability

Seeds mark the end of a plant’s reproductive cycle and the beginning of a new generation, representing the ability of higher plants to adapt and survive in changing, often hostile environmental conditions. They are not merely dormant propagules but highly active systems that incorporate genetic, physiological, and biochemical processes influenced by environmental cues. Seed physiology, including development, maturation, dormancy, germination, and vigour, is intricately affected by abiotic and biotic stresses. Environmental factors, including temperature, light, humidity, and oxygen availability, along with soil and microbial interactions, govern key transitions in the seed life cycle. Phytohormones, including abscisic acid (ABA), gibberellins (GA), and jasmonates (JA), coordinate these processes through complex signalling networks that regulate seed dormancy, germination, and resilience. In an era of increasing climate change, where temperature extremes, drought, and soil degradation threaten crop productivity and biodiversity, understanding how environmental stresses interact with seed physiology has become essential for maintaining agriculture and ecosystem stability (Kranner et al., 2010; Salvi et al., 2021, 2022; Kajal et al., 2023).

The interaction between stress and seed physiology involves tightly regulated molecular and metabolic adaptations. Seeds perceive environmental fluctuations and activate defence mechanisms, including the accumulation of protective metabolites, antioxidant activity, and membrane lipid remodelling (Kaur et al., 2024; Sonowal et al., 2024). These responses preserve cellular integrity during stress and desiccation, thereby sustaining seed viability and vigour. In addition, epigenetic and transcriptional regulation enables stress memory, allowing plants to adapt subsequent developmental responses. Understanding these mechanisms advances knowledge of plant stress adaptation and supports crop improvement strategies for enhanced stress tolerance and seed quality (Kaur et al., 2024). Collectively, the studies in this Research Topic provide integrated biochemical, developmental, ecological, and socio-economic insights into seed responses to environmental cues, with emphasis on how stress shapes seed development, performance, and resilience.

A study by Jha et al. investigated metabolomic and lipidomic alterations in heat-stressed chickpea seeds, providing molecular insight into heat-induced metabolic reprogramming. Chickpea, a key legume crop in semi-arid regions, suffers substantial reductions in yield and seed quality under high-temperature stress during reproduction. Using untargeted metabolomic and lipidomic profiling, the study identified metabolites and lipid signatures distinguishing heat-tolerant and heat-sensitive genotypes. These findings demonstrate that biochemical plasticity at the seed level is central to thermal resilience. The identification of potential biomarkers further supports breeding strategies aimed at improving heat tolerance and seed vigour, highlighting the complex interaction between environmental stress and seed physiology. Beyond biochemical adaptation, agricultural resilience also depends on effective seed systems that ensure access to quality planting material. Blalogoe et al. highlighted the influence of socio-economic and infrastructural constraints on seed quality and distribution. The study showed that farmer-managed systems supply more than 90% of okra seeds, whereas regulated systems, despite providing improved varieties, remain costly and geographically inaccessible. The authors proposed decentralised seed hubs, participatory varietal evaluation, and flexible certification frameworks to strengthen seed accessibility and resilience. These findings emphasise that the successful deployment of stress-tolerant varieties depends not only on physiological resilience but also on inclusive and adaptive seed systems.

Complementing these perspectives, Lopes et al. examined the transition from embryogenesis to seed filling in Phaseolus vulgaris, providing developmental insight into seed formation under environmental influence. Through integrated morphological, histological, and transcriptomic analyses, the study identified the embryogenesis-to-seed-filling transition between 10 and 14 days after anthesis, earlier than previously reported. This phase was characterised by the initiation of storage protein and starch accumulation alongside the upregulation of hormone-regulated genes. These findings refine the molecular framework governing seed composition and quality. Environmental stresses, including heat and nutrient imbalance, may disrupt this transition, thereby affecting seed viability and nutritional value. By defining key developmental and molecular milestones, the study offers valuable targets for breeding strategies aimed at improving developmental stability and environmental adaptability. Expanding the discussion to ecological and agronomic dimensions, Zhang et al. demonstrated that ecological management practices can mitigate environmental stress and improve seed outcomes. Intercropping cotton with cumin at optimal densities modified soil temperature, moisture, and salinity dynamics, resulting in enhanced soil quality and yield performance. Moderate intercropping density reduced soil electrical conductivity and weed pressure while increasing soil respiration and water-use efficiency. These improvements in the soil microenvironment positively influenced seed development and quality, highlighting the role of ecological design in buffering abiotic stress. The study underscores the importance of integrating agronomic management with seed–environment interactions to enhance agroecosystem sustainability and resilience. Li et al. investigated the role of BnaABI5 in Brassica napus and identified its involvement in regulating erucic acid biosynthesis. Although ABI5 is primarily associated with ABA signalling and plant development, the study revealed an additional regulatory function in seed oil metabolism. BnaABI5 and BnaFAE1, a key gene in erucic acid synthesis, exhibited coordinated expression during seed development and high seed-specific activity. Functional analyses showed that BnaABI5 acts as a nuclear-localised bZIP transcription factor, while BnaMED25 enhances its activation of BnaFAE1. Overexpression studies in both Arabidopsis and oilseed rape increased FAE1 expression and erucic acid accumulation in seeds. These findings establish a regulatory mechanism in which BnaABI5 and BnaMED25 jointly promote long-chain fatty acid biosynthesis, revealing a previously uncharacterised role of ABI5 in seed oil metabolism.

At a broader scale, Liu et al. conducted a bibliometric analysis of global and Chinese research on seed responses to environmental stress from 1975 to 2024. The study revealed sustained growth in this field, driven by technological advancements, climate change concerns, and agricultural policy priorities. International research primarily emphasised molecular mechanisms, including hormone signalling and oxidative stress, whereas Chinese studies focused more on applied agricultural challenges such as salinity, drought, heavy metal toxicity, and low-temperature stress. The analysis also identified fragmented collaboration networks and unequal research capacity among countries and institutions. The authors highlighted the need for stronger interdisciplinary and international collaboration, integrated studies on combined stresses, and sustainable innovations, including nanotechnology and stress-resilient crop breeding, to improve agricultural adaptation under climate change. Collectively, the studies in this Research Topic demonstrate that seed physiology is central to plant adaptation under environmental stress. Across metabolomics, developmental biology, agroecology, and socio-economic frameworks, seeds function as dynamic integrators of genetic potential, metabolic plasticity, and environmental signalling. Heat-stressed chickpea seeds reveal metabolic pathways associated with thermal resilience, while the okra seed system in Benin exposes socio-economic barriers limiting access to improved varieties. Research on *Phaseolus vulgaris* refines the molecular understanding of seed development, whereas cotton–cumin intercropping illustrates how ecological management can mitigate stress at the soil–seed interface. Similarly, studies in Brassica napus identify a novel role of ABI5 in seed oil metabolism. Together, these findings highlight the importance of integrated, multi-scale approaches for strengthening crop resilience in changing environments.

Overall, this Research Topic demonstrates that seed adaptation is governed by interconnected metabolic, hormonal, genetic, ecological, and socio-economic processes. As climate pressures intensify, understanding seed responses to environmental stress will remain essential for advancing crop improvement, ensuring food security, and sustaining resilient agricultural systems.
